# BRCA1 regulates PIG3-mediated apoptosis in a p53-dependent manner

**DOI:** 10.18632/oncotarget.3263

**Published:** 2015-02-18

**Authors:** Wenwen Zhang, Jiayan Luo, Fengxia Chen, Fang Yang, Wei Song, Aiyu Zhu, Xiaoxiang Guan

**Affiliations:** ^1^ Department of Medical Oncology, Jinling Hospital, Medical School of Nanjing University, Nanjing 210002, China; ^2^ Department of Medical Oncology, Jinling Hospital, School of Medicine, Southern Medical University, Guangzhou 510282, China

**Keywords:** PIG3, BRCA1, p53, prohibitin, breast cancer

## Abstract

BRCA1 plays a key role in the regulation of p53-dependent target gene transcription activation. Meanwhile, the p53 inducible gene 3 (*PIG3*) is a downstream target of p53 and is involved in p53-initiated apoptosis. However, little is known about whether BRCA1 can regulate PIG3-mediated apoptosis. Using a tissue microarray containing 149 breast cancer patient samples, we found that BRCA1 and PIG3 expression status were significantly positively correlated (*r* = 0.678, *P* < 0.001) and identified a significant positive correlation between high expression of BRCA1 and/or PIG3 and overall survival (OS). Moreover, we reveal that overexpression of BRCA1 significantly increased expression of PIG3 in cells with intact p53, whereas no increase in PIG3 was observed in p53-null MDA-MB-157 cells and p53-depleted HCT116p53^−/−^ cells. Meanwhile, ectopic expression of BRCA1 could not lead to an increase expression level of prohibitin (PHB), which we have previously identified to induce PIG3-mediated apoptosis. Finally, ChIP analysis revealed that PHB can bind to the PIG3 promoter and activate PIG3 transcription independent of p53, although p53 presence did enhance this process. Taken together, our findings suggest that BRCA1 regulates PIG3-mediated apoptosis in a p53-dependent manner, and that PIG3 expression is associated with a better OS in breast cancer patients.

## INTRODUCTION

BRCA1 is a tumor suppressor protein with important roles in multiple cellular processes, including DNA damage repair, cell cycle checkpoint control, transcriptional regulation, chromatin remodeling, and apoptosis [1–6]. The modulation of transcription factor activity by BRCA1 is best illustrated by its regulation of p53. BRCA1 (residues 224–500) interacts with p53 (residues 300–393), leading to increased transcription from p53-responsive promoters such as p21 and bax and induction of cancer cell apoptosis [7]. However, while BRCA1 can stimulate p53-dependent transcription, BRCA1-stabilized p53 is redirected to stimulate the transcription of genes involved with DNA repair and/or cell cycle arrest instead of pro-apoptotic genes [8].

The p53-inducible gene 3 (*PIG3*) was discovered during evaluation of serial analysis of gene expression patterns in a study designed to identify genes induced by *p53* before the onset of apoptosis [9]. The *PIG3* promoter contains a biding site for *p53* and transcription from it occurs prior to the onset of p53-initiated apoptosis [10]. The amino acid sequence of PIG3 is highly homologous to that of NADH quinine oxidoreductase 1 (NQO1), suggesting that PIG3 contributes to the generation of reactive oxygen species (ROS) [9]. In support of this, both *in vitro* and *in vivo* functional analyses have found that PIG3 generates ROS and can induce apoptosis [9, 11]. PIG3 has also been reported to mediate cancer cell death induced by glutathione peroxidase 3 (GPx3), with depletion of PIG3 or mutation of the PIG3 binding motif in GPx3 abrogating the increases in ROS and caspase-3 activity that are normally observed [12]. Recently, PIG3 has been shown to play an important role in the cellular response to DNA damage, such as in checkpoint signaling and DNA repair [13]. PIG3-depleted cells demonstrated increased sensitivity to DNA damage agents and a defective DNA repair phenotype [13]. Therefore, while it is considered a p53 dependent pro-apoptotic molecule, PIG3 is also involved in DNA repair. Moreover, whether BRCA1 regulates PIG3-mediated apoptosis in a p53-dependent manner is unknown. Additionally, silencing of *BRCA1* partially affects p53-dependent activation of PIG3 [8]. The present study therefore aims to investigate the signaling cascade linking p53 with PIG3 and assess its role in cellular apoptosis.

## RESULTS

### PIG3 and BRCA1 are associated with overall survival in breast cancer patients

**Figure 1:**
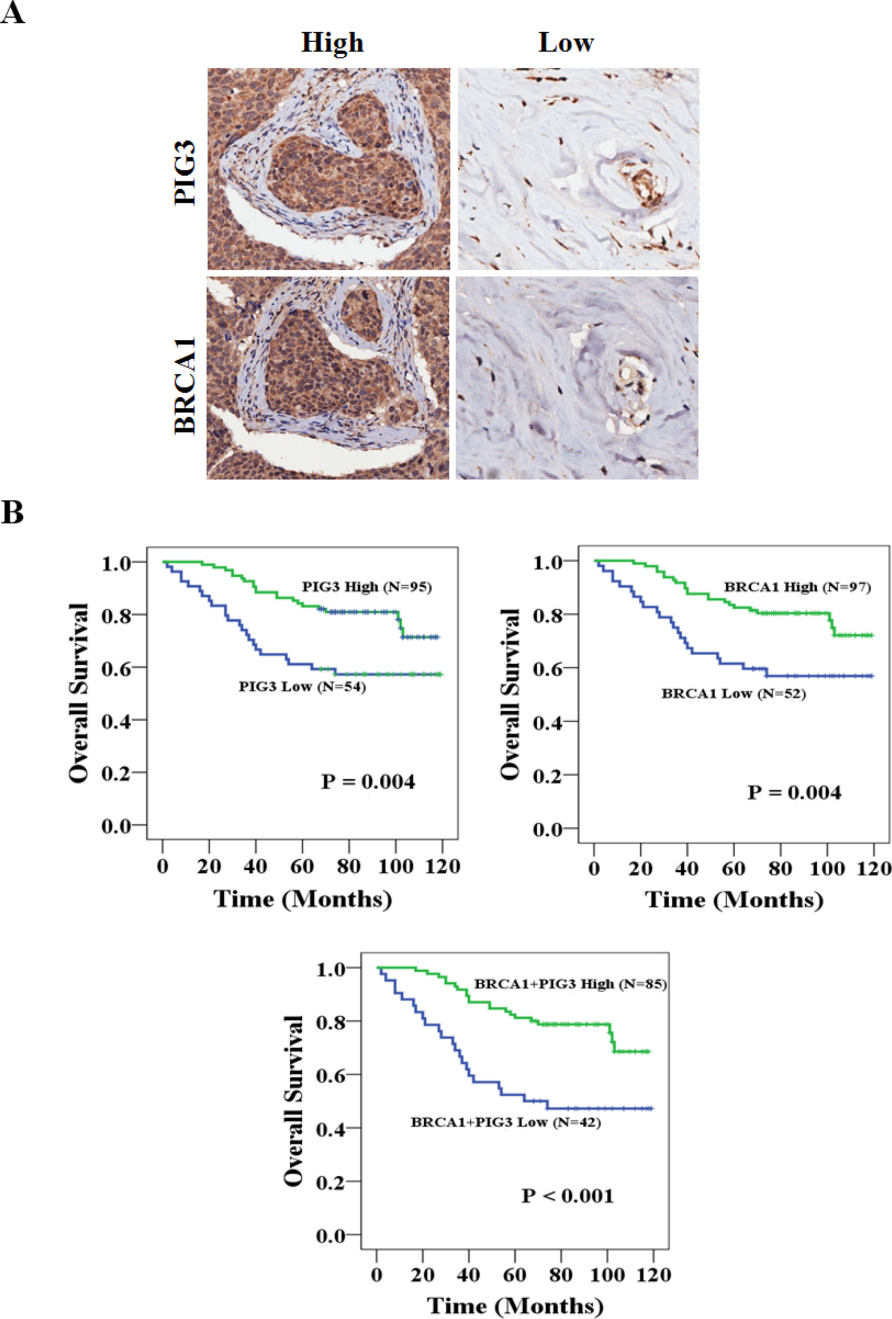
PIG3 and BRCA1 are associated with OS in breast cancer patients **(A)** High magnification (200×) regions shown immunohistochemical analysis of PIG3 and BRCA1 high or low expression breast cancer patient tissues. **(B)** High PIG3 and/or BRCA1 expression was associated with better OS (all *P* < 0.05).

To evaluate the putative association between PIG3 and BRCA1 with overall survival (OS) in human breast cancer, we performed immunohistochemical (IHC) staining of these proteins in malignant tumor samples from 149 patients using a tissue microarray (Figure 1A). High PIG3 expression was associated with better OS (Figure 1B, 102.08 vs. 81.10 months; *P* = 0.004) and high BRCA1 expression was also associated with better OS (102.40 vs. 81.15 months; *P* = 0.004). Moreover, OS was improved when the expressions of PIG3 and BRCA1 were both high (Figure 1B, 100.32 vs. 72.39 months; *P* < 0.001). Demographic, pathological, and clinical variables were collected and the correlation of these with PIG3 and BRCA1 expression was determined (Table 1). Of the 149 tumor tissues, 95 cases (63.8%) and 54 cases (36.2%) expressed PIG3 at high and low levels, respectively, while 97 cases (65.1%) and 52 cases (34.9%) expressed BRCA1 at high and low levels, respectively. Age, tumor size, and lymph nodal status were not significantly associated with PIG3 or BRCA1 expression (Table 1). We then determined whether a correlation between PIG3 and BRCA1 exists by using tumor microarrays (Table 2). A significant positive correlation between PIG3 and BRCA1 expression was identified using the breast cancer tissue-array (*r* = 0.678, *P* < 0.001). Taken together, these data suggest that PIG3 expression was positively associated with BRCA1 expression, and that high PIG3 and/or BRCA1 expression was associated with better OS in human breast cancer patients.

**Table 1:** Relationship between expression of BRCA1, PIG3 and clinicopathologic characteristics of breast cancer patients

Variables	BRCA1 high (*n* = 97)		BRCA1 low (*n* = 52)		*P*-value	PIG3 high (*n* = 95)		PIG3 low (*n* = 54)		*P*-value
	No.	%	No.	%		No.	%	No.	%	
Age					0.952					0.129
≤ 57 years	49	50.5	26	50.0		51	53.7	22	40.7	
> 57 years	48	49.5	26	50.0		44	46.3	32	59.3	
T stage					0.442					0.923
T1	22	22.7	16	30.8		23	24.2	15	27.8	
T2	58	59.8	31	59.6		58	61.1	31	57.4	
T3	12	12.4	3	5.8		9	9.5	6	11.1	
T4	1	1.0	1	1.9		1	1.1	1	1.9	
NA^*^	4	4.1	1	1.9		4	4.2	1	1.9	
N stage					0.168					0.058
N0	51	52.6	27	51.9		53	55.8	25	46.3	
N1	27	27.8	10	19.2		26	27.4	11	20.4	
N2	12	12.4	7	13.5		11	11.6	8	14.8	
N3	5	5.2	8	15.4		4	4.2	9	16.7	
NA^*^	2	2.1	0	0		1	1.1	1	1.9	

* NA: Not Available; All patients were M0 stage.

**Table 2:** Correlative analysis of the PIG3 expression with BRCA1 at tumor microarray

	Tumor microarray (*n* = 149)	
	BRCA1(Positive)	BRCA1(Negative)
PIG3(Positive)	85	10
PIG3(Negative)	12	42
r	**0.678**	
*P*	**< 0.001**	

### BRCA1 positively regulates PIG3 expression in a p53-dependent manner

**Figure 2:**
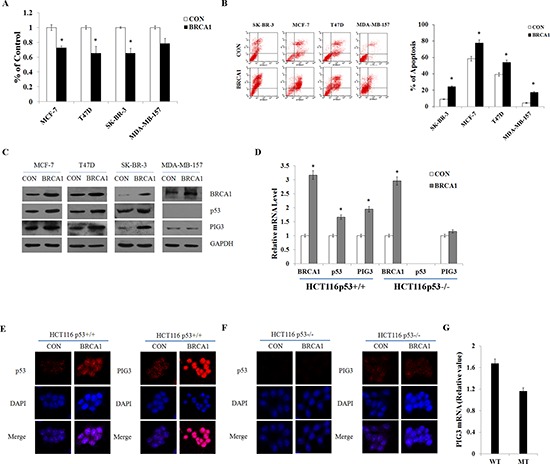
BRCA1 positively regulates PIG3 expression in a p53-dependent manner **(A** and **B)** Cell viability was measured by MTT assay, and apoptosis was detected by flow cytometry following transfection of plasmid encoding BRCA1 into MCF-7, T47D, SK-BR-3, and MDA-MB-157 cells. **(C)** BRCA1, p53, and PIG3 protein levels were determined by western blotting following transfection of plasmid encoding BRCA1 into MCF-7, T47D, SK-BR-3, and MDA-MB-157 cells, and normalized to GAPDH expression. **(D)** BRCA1, p53, and PIG3 mRNA expression levels were determined by RT-PCR following transfection of plasmid encoding BRCA1 into HCT116p53^+/+^ and HCT116p53^−/−^ cells, and normalized to GAPDH expression. **(E** and **F)** Localization and expression of p53 and PIG3 were determined by fluorescence microscopy following the same treatment as (D), with DMSO treatment as a control. Nuclei were stained with DAPI. The high magnification (200×) regions were shown above. **(G)** Relative levels of PIG3 mRNA in 206 breast cancer cell samples with no BRCA1 mutation (WT) and 392 breast cancer cell samples with a BRCA1 mutation (MT). Data analyzed using Oncomine (www.oncomine.org) from original published data (Garnett et al., 2012). Data are the mean of three independent experiments. *P < 0.05, as compared with untreated cells.

Mutations of the *BRCA1* gene confer a high risk for breast cancers [14]. Here, we found that in breast cancer cells, overexpression of BRCA1 inhibited cell proliferation and induced apoptosis in p53-expressing cell lines, while the effect of BRCA1 was attenuated in p53-null cells, MDA-MB-157 (Figure 2A and 2B) and validated in HCT116p53^−/−^ cells (Supplementary Figure S1A–S1C).

To investigate whether PIG3-mediated apoptosis could be regulated by BRCA1, plasmids encoding BRCA1 were transfected into p53 wild-type and p53-null cell lines and the levels of BRCA1, p53, and PIG3 mRNA and protein were assessed by RT-PCR and western blot analysis. Additionally, immunofluorescent staining was used to verify the expression and examine the subcellular localization of the proteins. Overexpression of BRCA1 significantly increased expression of both PIG3 and p53, consistent with previous studies reporting that BRCA1 could direct transcriptional co-activation of p53 and cooperatively induce apoptosis of cancer cells [7, 15]. However, BRCA1 transfection did not induce PIG3 expression in either the p53-null cell line MDA-MB-157, or the HCT116p53^−/−^ cell line (Figure 2C–2F). Furthermore, Analysis of a public database identified significantly reduced PIG3 mRNA levels in 392 breast cancer cell samples with a BRCA1 mutation, compared with 206 breast cancer cell samples without BRCA1 mutation (Figure 2G) [16]. Taken together, these data suggest that BRCA1 positively regulates PIG3 expression in a p53-dependent manner.

### BRCA1 induces PIG3 expression independent of *PHB*

**Figure 3:**
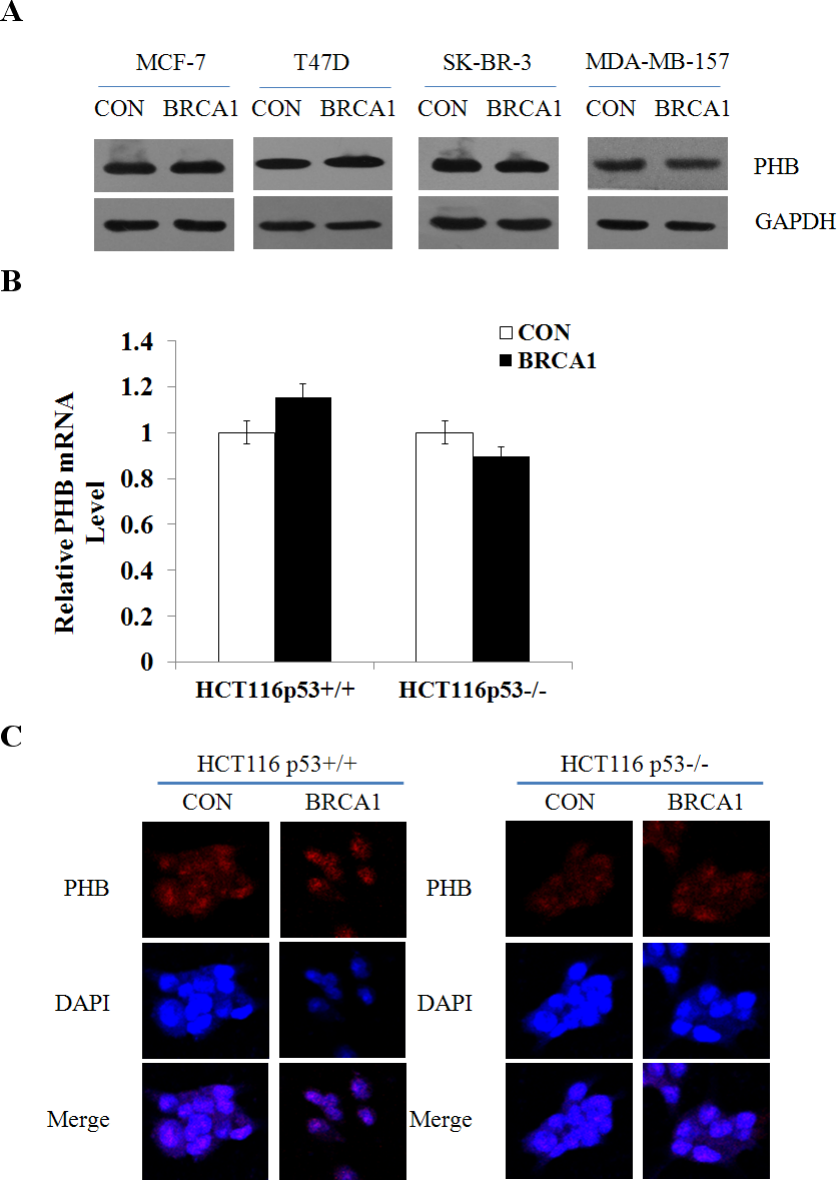
BRCA1 induces PIG3 expression independent of PHB **(A)** PHB protein levels were determined by western blotting following transfection of plasmid encoding BRCA1 into MCF-7, T47D, SK-BR-3, and MDA-MB-157 cells, and normalized to GAPDH expression. **(B)** PHB mRNA expression levels were determined by RT-PCR following transfection of plasmid encoding BRCA1 into HCT116p53^+/+^ and HCT116p53^−/−^ cells, and normalized to GAPDH expression. Data are the mean of three independent experiments. *P < 0.05, as compared with untreated cells. **(C)** Localization and expression of PHB were determined by fluorescence microscopy following the same treatment as (B), with DMSO treatment as a control. Nuclei were stained with DAPI. The high magnification (200×) regions were shown above.

Our previous studies have identified that prohibitin (PHB) could also contribute to PIG3-mediated apoptosis [17]. Based on our findings, we postulated that BRCA1 could induce PIG3 expression in a PHB-dependent manner. To test it, we detected the effect of BRCA1 on the expression of PHB in both p53 wild-type and p53-null cells. However, the protein level and mRNA level of PHB were both unchanged after BRCA1 vector transfection (Figure 3A and 3B; Supplementary Figure S2). The immunofluorescent staining also showed no change of the expression and subcellular localization of the protein (Figure 3C). Taken together, these data suggest that BRCA1 induces PIG3 expression independent of PHB.

### PHB regulates PIG3-mediated apoptosis in a p53-depentent or -independent manner

**Figure 4:**
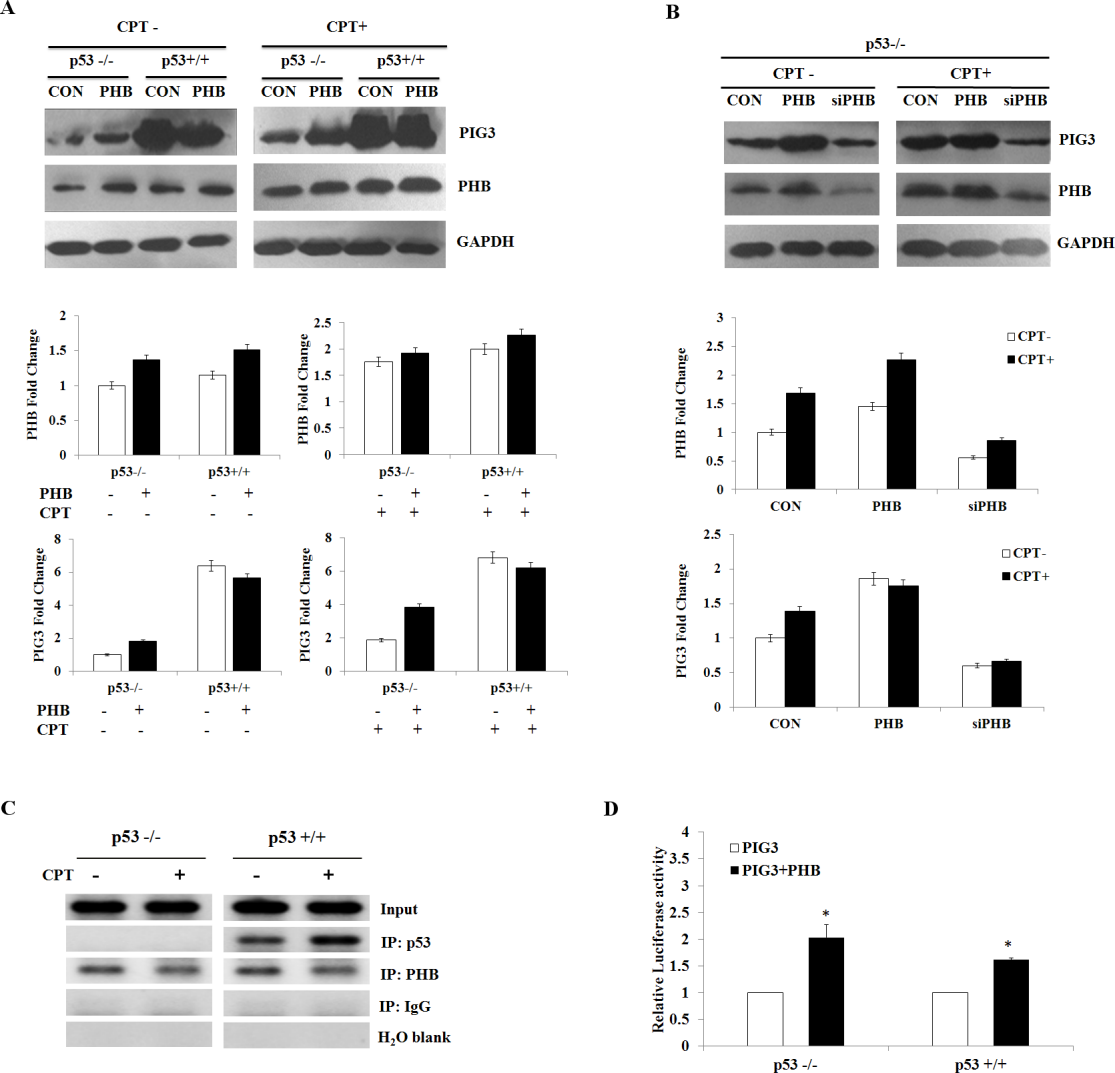
PHB regulates PIG3-mediated apoptosis in a p53-depentent or -independent manner **(A** and **B)** PIG3 and PHB protein levels were determined by western blotting and analyzed by grayscale software following transfection of plasmids encoding PHB or siPHB, into HCT116p53+/+ and HCT116p53^−/−^ cells in the presence or absence of 10 nM camptothecin. **(C)** ChIP assay for p53 and PHB binding of the PIG3 (TGYCC)_15_ motif in HCT116p53^+/+^ and HCT116p53^−/−^ cell lines in the presence or absence of camptothecin. **(D)** Plasmids encoding PIG3 were co-transfected into HCT116p53^+/+^ and HCT116p53^−/−^ cells with or without PHB. At 24 h post-transfection, the cells were subjected to luciferase assay. For comparison, the luciferase activity of the ncRNA-transfected cells was set as 1. Data are the mean of three independent experiments. *P < 0.05, as compared with untreated cells.

Our previous studies have reported that the PIG3 promoter motif (TGYCC)_15_ is required for effective transcriptional activity of the promoter, and that PHB could contribute to PIG3-mediated apoptosis [18, 19]. To further investigate the roles of PHB and p53 in PIG3-mediated apoptosis, we examined the expression of PIG3 and PHB in HCT116p53^+/+^ and HCT116p53^−/−^ cells overexpressing PHB in the presence or absence of camptothecin (CPT), a topoisomerase 1 inhibitor and an effective apoptotic stressor [20]. Protein expression of PIG3 was elevated in the HCT116p53^+/+^ cells compared with HCT116p53^−/−^ cells, regardless of CPT treatment (Figure 4A). Following PHB overexpression, expression of PIG3 was increased in HCT116p53^+/+^ cells, while PIG3 was also significantly increased in the absence of p53 in HCT116p53^−/−^ cells transfected with PHB. To investigate whether PHB could regulate the expression of PIG3 in a p53-independent manner, we also examined the effect of PHB depletion by RNA interference on the expression of PIG3. Transfection of HCT116p53^−/−^ cells with PHB-targeting shRNA constructs efficiently suppressed PHB expression and inhibited expression of PIG3 (Figure 4B). Similarly, when the PHB-depleted cells were exposed to 10 nM camptothecin, PIG3 down-regulation was observed (Figure 4B).

To determine whether PHB interacts with the *PIG3* promoter in the absence of p53, we performed chromatin immunoprecipitation (ChIP) assays to measure the relative binding of PHB to the (TGYCC)_15_ motif of the PIG3 promoter in both HCT116p53^+/+^ and HCT116p53^−/−^ cells. PHB specifically bound to the PIG3 (TGYCC)_15_ motif *in vivo*, regardless of p53 status and camptothecin treatment (Figure 4C). Furthermore, co-transfection of plasmids encoding PHB and PIG3 into HCT116p53^+/+^ or HCT116p53^−/−^ cells harboring the PIG3 promoter luciferase construct significantly increased luciferase activity compared with cells overexpressing PIG3 alone (Figure 4D). Additionally, overexpression of PHB in both HCT116p53^+/+^ and HCT116p53^−/−^ cell lines led to G_1_/S cell cycle arrest (Supplementary Figure S3A–S3B). Collectively, these results strongly suggest that PHB regulated the expression of PIG3 in a p53-depentent or -independent manner.

## DISCUSSION

*PIG3* was originally identified in a screen for genes induced by p53 before the onset of apoptosis [9]. Later, PIG3 was found to suppress catalase activity through direct binding of the enzyme, leading to increased ROS generation in response to DNA damage [21]. Lee et al. have also reported that PIG3 knockdown causes defects at the intra-S phase and G_2_/M DNA damage checkpoints. Furthermore, silencing of PIG3 leads to increased sensitivity to DNA damage agents, including UV and radiomimetic drugs, and contributes to the recruitment of 53BP1, Mre11, Rad50, and Nbs1 to sites of DNA double-strand breaks (DSBs) [13]. It is well known that BRCA1 plays a critical role in the regulation of homologous recombination (HR)-mediated DNA DSB repair [22]. BRCA1 interacts with the Mre11/Rad50/Nbs1 complex to regulate end resection activity, and contributes to the activation of HR-mediated DSB repair in the S and G_2_ phases of the cell cycle [23].

An interaction between BRCA1 and p53 results in the redirection of p53-mediated gene transactivation from pro-apoptotic targets to genes involved in DNA repair and/or cell cycle arrest [8]. Given the roles of PIG3 and BRCA1 in regulating apoptosis and the DNA damage response, we suspected that PIG3 might have an additional connection with BRCA1 in mediating PIG3-induced apoptosis. Our previous studies have shown that BRCA1 positively regulates PIG3 expression in a p53-dependent manner. In p53-null MDA-MB-157 cells and p53-depleted HCT116p53^−/−^ cells, BRCA1 failed to induce PIG3 expression [24, 25]. Recent study has reported that BRCA1 is exported to the cytoplasm following DNA damage in a p53-dependent manner and p53 mediates BRCA1 nuclear export via protein-protein binding, while augmentation of cytosolic BRCA1 significantly enhances cancer cell susceptibility to ionizing radiation [26]. We suspected that dysfunctional p53 compromises nuclear export of BRCA1 as a mechanism to attenuate the regulation of PIG3 expression by BRCA1, which remain to be confirmed by further studies.

Tissue microarray analysis of 149 breast cancer patient samples revealed a positive correlation between PIG3 and BRCA1 expression (*r* = 0.678, *P* < 0.001). High PIG3 and/or BRCA1 expression was also associated with better OS in human breast cancer patients [27]. Building upon the previously established roles of BRCA1 and PIG3 in breast cancer, our results provide the first explanation of an important mechanism for BRCA1 to positively regulate the expression of PIG3 and influence the progression of breast carcinoma cells.

The promoter of PIG3 contains a variable number of tandem repeats (VNTRs) of pentanucleotides (TGYCC)_n_ that is known as a *p53* binding site [10]. Our previous study identified direct interactions of PHB and/or prohibiton (PHB2) with the (TGYCC)_15_ motif, using ligand-chromatography combined with liquid chromatography-tandem mass spectrometry analyses [19]. Functional studies have suggested that PHB was capable of physically interacting with p53 *in vivo* and *in vitro*, and PHB was found to enhance p53-mediated transcriptional activity by enhancing its recruitment to promoters [28]. Therefore, it is conceivable that immediate p53 binding at the PIG3 promoter and subsequent interaction with PHB together may facilitate the p53-meidiated PIG3 transcriptional regulation. However, the data presented here that PHB was associated with the (TGYCC)_15_ motif *in vivo* regardless of p53 status or apoptotic stress. We also found that PHB upregulated PIG3 expression independent of p53, although p53 enhanced this process. Furthermore, knockdown of PHB inhibited camptothecin-induced apoptosis. The present study, together with previous findings in other laboratories, provides new evidence to explain the elusive functions of PHB, which have been considered both p53-dependent and independent based on the origin of PHB as a cellular inner membrane protein [29].

**Figure 5:**
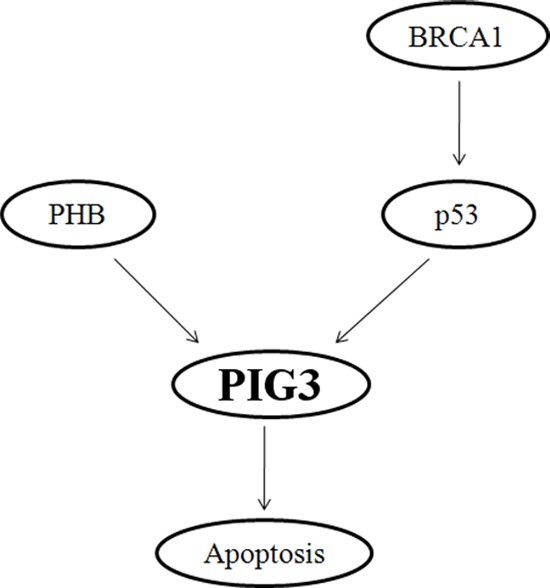
Schematic model depicting the critical role of BRCA1 in the regulation of PIG3-mediated apoptosis

Collectively, our results to date permit construction of a schematic model demonstrating the critical role of BRCA1 in the regulation of PIG3-mediated apoptosis (Figure 5). Most importantly, key features of this pathway are manifested in clinical breast cancer patients. Importantly, our data reveal that BRCA1 positively regulates PIG3 expression in a p53-dependent manner, and the clinical data demonstrate a statistically significant positive correlation between PIG3 and BRCA1 expression, with high PIG3 and/or BRCA1 expression were associated with better OS in human breast cancer patients. Together, our results provide a mechanism for how BRCA1 regulates PIG3 expression and illustrate the relevance of this mechanism to clinical outcomes in breast cancer patients. However, additional larger studies and more detailed investigations of the regulatory mechanisms underlying the influence of BRCA1 on the expression of PIG3 are needed to validate our findings.

## MATERIALS AND METHODS

### Clinical samples

Breast cancer tissues section containing HBre-Duc150Sur-02 (150 cancer cases) were provided by Outdo Biotech (Shanghai, China). Experiments were approved by the Ethics Committee of Jinling Hospital and were conducted in compliance with the Helsinki Declaration. Histological parameters were determined in accordance with the criteria of the World Health Organization. Pathologic staging was performed in accordance with the current International Union against Cancer tumor-lymph node metastasis classification.

### Immunohistochemistry

One hundred and forty-nine breast tumor tissue samples were deparaffinized in xylene, followed by heat-mediated antigen retrieval using citrate buffer (BioGenex Laboratories, San Ramon, CA, USA). Antibody staining was visualized with DAB (Sigma, D-5637) and hematoxylin counterstain. The percentage of positively stained cells was scored on a scale of 0 to 4 as follows: 0 (<1%), 1 (1–24%), 2 (25–49%), 3 (50–74%), and 4 (75–100%). The staining intensity was scored from 0 to 3 as follows: 0 (negative), 1 (weak), 2 (moderate), and 3 (strong). The scores for percentages of positive cells and staining intensities were then multiplied to generate an immunoreactivity score (IS) for each case. The IS ranged from 0–12 (0, 1, 2, 3, 4, 6, 8, 9, and 12). Cutoff values for this scoring system were assigned as follows: high expression of PIG3 and BRCA1 were defined as an IS of ≥ 4 (4, 6, 8, 9, and 12); and low expression was defined as an IS of < 4 (0, 1, 2, and 3). Immunohistochemical scoring was performed without prior knowledge of the clinical response. Immunostained sections were scanned using a microscope (Aiovert 200; Carl Zeiss).

### Cell lines and cell culture

The HCT116 human colon cancer cell lines (p53^+/+^ and p53^−/−^) were generously provided by Dr. Bert Vogelstein (Johns Hopkins University) [19]. Breast cancer cell lines MCF-7, T47D, SK-BR-3, and MDA-MB-157 were purchased from the American Type Culture Collection (Manassas, VA, USA). Cells were grown in RPMI 1640, DMEM or McCoy's 5A medium (GIBCO, Gaithersburg, MD, USA) supplemented with 10% fetal bovine serum (FBS) and 1% penicillin/streptomycin at 37°C in a humidified atmosphere containing 5% CO_2_.

### Plasmids and transient transfection

Plasmids pBABEpuro HA Brca1 (Plasmid #14999), pBK-CMV Pig3 (Plasmids #16496) were purchased from Addgene (USA). The vector pGPU6/GFP/Neo used for cloning PHB short hairpin RNA (shRNA) was purchased from GenePharma (Shanghai, China). The target sequences used for shRNA are listed in Supplementary Table S1. Generated plasmids were named siPHB1, siPHB2, siPHB3, and one with the highest targeting efficiency was chosen for further studies. A random sequence was used as negative control. Cells (1 × 10^6^ cells/well) were plated in 6-well plates 24 h prior to transfection. Plasmids were then transfected into cells using TurboFect Transfection Reagent (Thermo Scientific) according to the manufacturer's protocol. Following incubation at 37°C for 24 h, cells were collected and lysed to verify the expression of related proteins by western blot analysis.

### Cell survival (MTT) assay

A total of 1 × 10^4^ cells per well were seeded into 96-well plates and incubated with various concentrations of resveratrol or transfected with required plasmids for 48 h. Following addition of 20 μL of 0.5 mg/mL MTT solution (Sigma) to each well, the medium was replaced with 200 μL DMSO after 4 h and vortexed for 10 min. Absorbance was measured at 490 nm with a microplate reader (BIO-RAD, USA) to determine the relative numbers of viable cells. Assays were performed independently three times.

### Cell cycle and apoptosis analysis

Cells were treated with plasmids for 48 h, then harvested by trypsinization (no EDTA) and washed with phosphate-buffered saline (PBS). Analysis of the cell cycle and apoptosis was performed as previously described [30]. Each sample was tested in triplicate and untreated cells were used as controls.

### RNA isolation and quantitative RT-PCR

Total RNA was extracted from cultured cells using Trizol (Invitrogen, CA, USA) according to the manufacturer's protocol. Reverse transcription was conducted using a PrimeScript 1st Strand cDNA synthesis kit (TaKaRa, Dalian, China) according to the manufacturer's instructions. The sequences of the primers used for PCR are listed in Supplementary Table S2. PCR analysis was performed in a 20-μL volume with amplification conditions: 95°C for 2 min [94°C for 10 s, 59°C for 10 s, and 72°C for 40 s], 40 cycles. All reactions were performed in triplicate. Threshold cycles (CT) were determined using fixed threshold settings. All PCR assays included no template controls and were run in triplicate.

### Western blotting

Total protein was extracted using RIPA buffer supplemented with protease and phosphatase inhibitors, with protein concentrations determined using a BCA kit (Thermo Scientific). Approximately 20 μg protein was loaded per lane and separated on a sodium dodecylsulfate-polyacrylamide gel and blotted onto nitrocellulose. Blots were blocked with 5% dry milk in tris-buffered saline/0.1% tween-20 and incubated overnight with a diluted solution of primary antibody at 4°C, followed by incubation with a horseradish peroxidase-conjugated secondary antibody (1:5000) for 2 h. Antibodies used for western blot were: rabbit anti-BRCA1 antibody (CST9010), rabbit anti-p53 antibody (ab32049), rabbit anti-PIG3 antibody (ab64798, sc-30068), and rabbit anti-PHB antibody (Ls-B7282). Bands were normalized to GAPDH expression, which was used as an internal loading control. Results from at least three separate experiments were analyzed.

### Immunofluorescence analysis

Immunofluorescence staining was used to verify protein expression and examine the subcellular localization of p53, PHB, and PIG3. Cells were plated onto glass coverslips in 6-well plates, washed with PBS and fixed in 4% paraformaldehyde for 20 min, permeabilized with 0.1% TritonX-100 for 10 min, and incubated for 1 h at 37°C with the following antibodies: rabbit anti-p53 antibody (1:100, ab32049), rabbit anti-PHB antibody (1:100, Ls-B7282), and rabbit anti-PIG3 antibody (1:100, ab64798). Cells were then washed with PBS and incubated for 30 min at 37°C with mouse anti-rabbit IgG conjugated with FITC (Invitrogen; 1:200). Subsequently, nuclei were counterstained with 4′, 6-diamidino-2-phenylindole (DAPI; Sigma) for 10 min. Finally, cells were mounted with mounting solution (DAKO, Glostrup, Denmark) and examined under a LSM510 confocal microscope (Carl Zeiss, Gottingen, Germany).

### Dual-luciferase reporter assay

Cells (1 × 10^5^/well in a 6-well plate) were transiently transfected with 1 μg of luciferase construct (pGL3-promoter-luc, and pGL3-Luc) and 0.1 μg of pRL-Tk (Promega) together with the indicated plasmids using Lipofectamine/plus reagent (Invitrogen). After 24 h, cells were harvested using the Dual-luciferase assay system (Promega) according to the manufacturer's instructions, and the luciferase activities of cell extracts measured using a luminometer (Centro XS3 LB960, Berthold, Germany). Firefly luciferase activity was normalized to Renilla luciferase activity and relative activity expressed as fold induction. Each assay was performed in triplicate and the experiment was repeated at least three times.

### Chromatin immunoprecipitation assays

The ChIP assay was performed with a kit (Beyotime, China) according to the manufacturer's instructions. Briefly, 70% confluent HCT116p53^+/+^ and HCT116p53^−/−^ cells were treated with DMSO or 250 nM camptothecin for 24 h and then fixed in 1% formaldehyde for 15 min. Cells were lysed, and nuclei were pelleted by centrifugation. Nuclei were resuspended and sonicated on ice using a sonicator to shear the cross-linked DNA to an average length of 200–1000 bp and centrifuged at 12,000 rpm to remove insoluble material. Sheared chromatin was immunoprecipitated with 1 μg of anti-PIG3 (Santa Cruz Biotech, USA) or control IgG antibody overnight at 4°C. Cross-links were reversed by incubation with proteinase K in ChIP Elution buffer for 1 h at 62°C. The sequences of the PCR primers used are listed in Supplementary Table S3.

### Statistical analysis

SPSS Statistics 19.0 (SPSS Inc.) was used for statistical analysis. Data were analyzed using one-way ANOVA or a Student's *t*-test. Data are presented as means ± SD of three independent experiments. The log-rank test was used to assess statistical significance of Kaplan–Meier plots. The chi-square test was used for IHC data. The correlation between BRCA1 and PIG3 expression level was estimated by using the Spearman's correlation analysis, *P < 0.05 or **P < 0.001

## SUPPLEMENTARY FIGURES AND TABLES


