# Supplementary material for: BRCA1 regulates PIG3-mediated apoptosis in a p53-dependent manner
Source: Oncotarget. 2015 Feb 18;6(10):7608–18. doi: 10.18632/oncotarget.3263 (PMC4480703; doi:10.18632/oncotarget.3263)
Supplement: Supplementary file 1 [file oncotarget-06-7608-s001.pdf]

SUPPLEMENTARY FIGURES AND TABLES

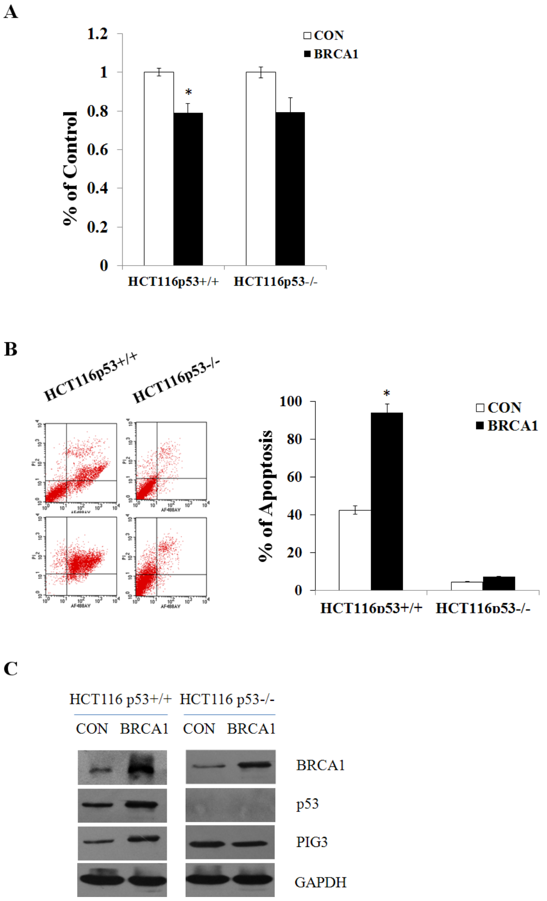

Supplementary Figure S1: BRCA1 positively regulates PIG3 expression in a p53-dependent manner. (**A** and **B**) Cell viability was measured by MTT assay, and apoptosis was detected by flow cytometry following transfection of plasmid encoding BRCA1 into HCT116p53$^{+/+}$ and HCT116p53$^{-/-}$ cells. (**C**) BRCA1, p53, and PIG3 protein levels were determined by western blotting following transfection of plasmid encoding BRCA1 into HCT116p53$^{+/+}$ and HCT116p53$^{-/-}$ cells, and normalized to GAPDH expression. Data are the mean of three independent experiments. *$P < 0.05$, as compared with untreated cells.

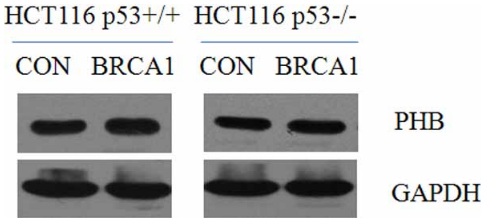

Supplementary Figure S2: BRCA1 induces PIG3 expression independent of PHB. PHB protein levels were determined by western blotting following transfection of plasmid encoding BRCA1 into HCT116p53$^{+/+}$ and HCT116p53$^{-/-}$ cells, and normalized to GAPDH expression. Data were represented as the average of three independent experiments.

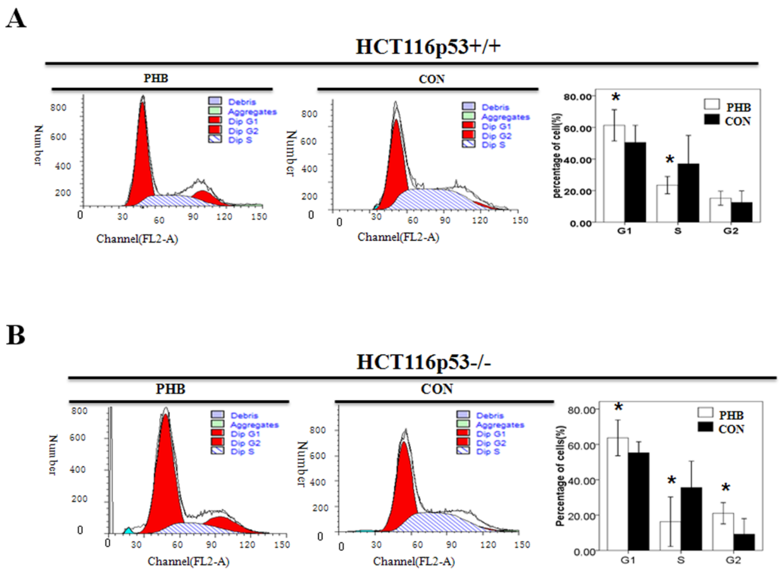

Supplementary Figure S3: PHB causes G1 cell cycle arrest regardless of p53 status. The HCT116p53$^{+/+}$ **(A)** and HCT116p53$^{-/-}$ **(B)** cells were transfected PHB vectors for 48 h, then cell cycle distribution was detected by flow cytometry analysis using PI staining. Data were represented as the average of three independent experiments. *$P < 0.05$.

Supplementary Table 1: Target sequences for shRNA

Gene	Target sequences
**PHB-homo-647**	5′- GAAGCAGAGAGGGCCAGATTT-3′
**PHB-homo-1453**	5′- GGAAACAGGTGTGTGTGAACT-3′
**PHB-homo-1682**	5′- GCTGAACATGCCTGCCTGCCAAAGA-3′
**Human GAPDH**	5′- GTATGACAACAGCCTCAAG-3′
**Negative Control**	5′- GTTCTCCGAACGTGTCACGT-3′

Supplementary Table 2: Primer sequences for quantitative RT-PCR

Gene	Sequences
BRCA1-F	5′- GGCTACAGAAACCGTGCCAA-3′
BRCA1-R	5′- CCGCTGCTTTGTCCTCAGAG-3′
p53-F	5′- AGATAGCGATGGTCTGGCCC-3′
p53-R	5′- AACCTCAGGCGGCTCATAGG-3′
PHB-F	5′- TTTGACTGCCGTTCTCGACC-3′
PHB-R	5′- AAGATGCGAGGAAGCTGGCT-3′
PIG3-F	5′- CCATGCAGGACTGAGTGGTG-3′
PIG3-R	5′- CTGCTCCAAGCTTTTCTGCC-3′
GAPDH-F	5′- CTGCCAACGTGTCAGTGGTG-3′
GAPDH-R	5′- TCAGTGTAGCCCAGGATGCC-3′

F: Forward; R: Reverse

Supplementary Table 3: The sequences of the PCR primers for PIG3 ChIP

Gene	Sequences
PIG3-Forward	5′- GGGCGCTGCGGTGCCAGCCTGAG-3′
PIG3-Reverse	5′- ACCTTCAGGAGGACTTCACC-3′
β-actin-Forward	5′- TCACCCACACTGTGCCCATCTACGA-3′
β-actin-Reverse	5′- CAGCGGAACCGCTCATTGCCAATGG-3′
